# 环状RNA在肿瘤免疫治疗中的研究进展

**DOI:** 10.3779/j.issn.1009-3419.2021.101.31

**Published:** 2021-10-20

**Authors:** 宸瑜 张, 垒 彭, 敬斌 纪, 文捷 矫

**Affiliations:** 266071 青岛，青岛大学附属医院胸外科 Department of Thoracic Surgery, the Affiliated Hospital of Qingdao University, Qingdao 266071, China

**Keywords:** 环状RNA, 肿瘤, 肺肿瘤, 肿瘤免疫治疗, 免疫检查点, Circular RNAs, Tumor, Lung neoplasms, Tumor immunotherapy, Immune checkpoint

## Abstract

肿瘤免疫治疗是近年来发展起来的全新治疗方法，极大地改变了癌症的治疗方案，为癌症患者带来了新的选择，但并不是所有患者在应用免疫治疗后都疗效明显，因此如何更加有效地选择适用免疫治疗的患者和提高免疫治疗疗效是十分值得探讨的问题。随着对环状RNA（circular RNAs, circRNAs）不断深入研究发现，circRNA不仅在肿瘤标志物、肿瘤进展和预后领域中作用显著，还可以在多种肿瘤中异常表达，进而通过多种途径影响肿瘤免疫。因此检测circRNA的表达量不仅可能作为筛选适用免疫治疗的患者的补充手段，还可能用于预测肿瘤免疫治疗的疗效。本文总结了circRNA在以肺癌为首的多种肿瘤免疫治疗中的研究进展，并讨论了其在肿瘤免疫治疗中潜在的作用，意在为circRNA的临床应用提供参考。

## Abstract (en)

Tumor immunotherapy is a new therapy which developed in recent years, it has greatly changed the therapeutic schedules and brought new options for patients. However, not all patients can have obvious therapeutic effects after using immunotherapy. So selecting more suitable patients and raising immunotherapy effect are worthy to discuss. With the research of circular RNAs (circRNAs), circRNAs have been found that they not only play a significant role in the field of tumor markers, tumor progression and prognosis, but also can abnormally express in a variety of tumors and affect tumor immunity. Therefore, the circRNAs expression may not only can be used as a supplementary method for selecting patients, but also can be used to predict the efficacy of tumor immunotherapy. In this article, we summarize current knowledge on circRNAs abnormally expressed in many cancers, especially lung cancer which can affect tumor immunity, and discuss its potential effects in tumor immunotherapy, and we hope to provide more references for the clinical practice of circRNAs.

1976年Sanger博士在Viroids中第一次发现环状RNA（circular RNAs, circRNAs）^[1]^，在之后的一段时间内，circRNA普遍被认为是由于剪接错误产生的副产物，并不具有已知的生物功能^[2]^。近些年来，得益于RNA测序技术的不断更新和进步，目前已经有成千上万种circRNA被发现并引起人们的重视与深入研究。CircRNA是一种单链共价闭合的环状RNA分子，通常由前体核糖核酸反向剪接而来，下游5’端剪接位点（剪接供体）和上游的3’端剪接位点（剪接受体）相连接从而产生首尾相连的circRNA^[3]^，也正因为如此，circRNA比线性RNA更能够抵抗核酸外切酶降解从而比线性RNA更加稳定^[4]^。CircRNA的功能主要包括提供微小核糖核酸（microRNA, miRNA）结合位点从而充当miRNA海绵、调控基因的转录、与蛋白质结合、作为蛋白质翻译模板等^[2]^。研究^[5]^发现在不同细胞和组织中，不同circRNA的表达是不相同的，即具有组织特异性，大多数circRNA在不同的疾病和肿瘤中往往异常表达，这使通过circRNA来鉴别不同的疾病和肿瘤成为了可能。除此之外，circRNA的异常表达可能会影响肿瘤的发生与进展，因此通过干预circRNA表达或抑制其功能的方法可能用于治疗或者辅助治疗肿瘤和疾病。

肿瘤免疫治疗方法主要包括免疫检查点抑制剂疗法、癌症疫苗、过继性免疫治疗和溶瘤病毒等。自William Coley首次利用Coley毒素激活免疫系统治疗癌症，从而开创肿瘤免疫治疗先河以来^[6]^，肿瘤免疫治疗取得了长足进展。2011年以及2014年细胞毒性T淋巴细胞相关蛋白4（cytotoxic T lymphocyte-associated antigen-4, CTLA-4）抗体伊匹单抗（Ipilimumab）和程序性细胞死亡受体1（programmed cell death-1, PD-1）抗体帕博利珠单抗（Pembrolizuamb）获得批准可被用于黑色素瘤的治疗，标志着肿瘤免疫检查点疗法时代的开始^[7]^。肿瘤免疫治疗是目前肿瘤治疗领域的研究热点，目前已经有研究^[8]^表明circRNA可以在肿瘤免疫治疗中起到一定作用。CircRNA主要作为一种竞争性内源RNA（competing endogenous RNA, ceRNA）或miRNA海绵发挥功能，通过利用结合位点与miRNA竞争性结合，从而阻碍miRNA与下游的mRNA结合，最终阻碍其原有的功能从而影响肿瘤免疫；少数研究中circRNA通过与RNA聚合酶II结合形成复合体直接调控基因的转录从而影响肿瘤的免疫，例如过表达的circRNA CDR1-AS可使趋化素样因子超家族4（CKLF-like MARVEL transmembrane domain 4, CMTM4）和趋化素样因子超家族6（CKLF-like MARVEL transmembrane domain 6, CMTM6）mRNA表达水平增加，最终导致PD-L1表达水平增加。本文将对circRNA在以肺癌为首的肿瘤免疫治疗研究中的进展作一综述。

## 1 CircRNA与免疫检查点疗法

由T细胞介导的抗肿瘤免疫是目前肿瘤免疫治疗的热点，CTLA-4和PD-1是T细胞表面的共抑制受体，当其分别与相对应的配体结合后，T细胞的抗肿瘤活性将受到抑制^[9]^，从而使肿瘤细胞得以逃避免疫系统的杀伤。通过使用免疫检查点抑制剂可以激活T细胞的抗肿瘤活性，进而消灭肿瘤细胞^[10]^。目前已经有多项关于circRNA影响CTLA-4、PD-1、程序性死亡配体-1（programmed cell death ligand-1, PD-L1）等的表达从而影响肿瘤免疫逃逸的研究，本综述主要描述目前比较常见的免疫检查点与对应的circRNA之间的联系及可能的影响，所涉及的circRNA总结于表 1。

**1:** 环状RNA与免疫检查点疗法 CircRNA and immune checkpoint therapy

CircRNA	Tumor	Immune checkpoint	Effect of tumor immunity^a^
Circ-UBAP2	PAAD	CTLA-4，PD-1	Suppression
CircFGFR1	NSCLC	PD-1	Suppression
CircRNA-002178	LUAD	PD-1, PD-L1	Suppression
Circ-0020710	Melanoma	PD-1	Suppression
Circ-0001068	OC	PD-1	Suppression
Circ-CPA4	NSCLC	PD-L1	Suppression
Circ-0000284	NSCLC	PD-L1	Suppression
CircCHST15	LC	PD-L1	Suppression
Has-circ-0000190	LC	PD-L1	Suppression
CircKRT1	OSCC	PD-L1	Suppression
Circ-KRT6C	CRC	PD-L1	Suppression
Has-circ-0003288	HCC	PD-L1	Suppression
CircRNA CDR1-AS	Colon cancer	PD-L1	Suppression
^a^: Tumor immune effects when circRNAs are overexpressed. PAAD: pancreatic adenocarcinoma; NSCLC: non-small cell lung cancer; LUAD: lung adenocarcinoma; OC: ovarian cancer; HCC: hepatocellular carcinoma; LC: lung cancer; OSCC: oral squamous cell carcinoma; CRC: colorectal cancer; PD-1: programmed cell death-1; PD-L1: programmed cell death ligand-1; CTLA-4: cytotoxic T lymphocyte-associated antigen-4.			

### 1.1 CircRNA影响CTLA-4的表达

CTLA-4（CD152）是第一代免疫检查点的靶标，属于免疫球蛋白超家族，通常以低水平表达，并且在细胞活化的早期阶段有着重要的调节作用^[11]^。CTLA-4作为一种在T淋巴细胞膜表面发挥功能的免疫调节受体，却主要存在于细胞内囊泡中，通过内吞作用形成循环与降解^[12, 13]^，在与其配体结合后可以负性调节免疫，从而抑制T细胞的抗肿瘤活性。有研究团队^[14]^通过生信分析发现高表达的circ-UBAP2在胰腺癌中可以抑制has-miR-494的表达从而促进胰腺癌的进展，同时通过分析发现趋化因子受体4（C-X-C motif chemokine receptor 4, CXCR4）、多配体蛋白聚糖1（syndecan 1, SDC1）、锌指结合蛋白1（zinc finger E-box binding homeobox 1, ZEB1）和低氧诱导因子-1α（hypoxia-inducible factor 1 subunit alpha, HIF1α）是circ-UBAP2/has-miR-494 ceRNA网络的潜在靶标，且既往已有其他研究发现miR-494可以抑制CXCR4的表达，研究团队还发现CXCR4与ZEB1的表达和CTLA-4与PD-1的表达呈正相关，表明CXCR4和ZEB1的高表达可能会导致CTLA-4与PD-1表达升高从而促进肿瘤免疫逃逸，因此circ-UBAP2有可能通过调控has-miR-494进而调控CXCR4和ZEB1的方式，最终调控CTLA-4与PD-1的表达，从而抑制抗原呈递并促进胰腺癌细胞的免疫逃逸，使肿瘤细胞逃脱免疫系统的杀伤，使肿瘤进一步发展。

### 1.2 CircRNA影响PD-1的表达

PD-1是一种由288个氨基酸构成的I型跨膜糖蛋白，属于免疫球蛋白超家族，是一种重要的免疫抑制分子。其结构由包含一个IgV结构域的胞外结构域、疏水性跨区和胞质区构成^[15]^。胞质尾部有由两个酪氨酸残基分别构成的免疫受体酪氨酸抑制基序（immune-receptor tyrosine-based inhibitory motif, ITIM）和免疫受体酪氨酸转换基序（immune-receptor tyrosine-based switch motif, ITSM），ITSM内的酪氨酸残基磷酸化从而使PD-1介导的免疫抑制发挥作用，故PD-1在免疫应答中有着重要的负性调控作用^[16]^。

对于circRNA在PD-1免疫检查点疗法中的作用而言，circRNA主要通过提供结合位点，即作为miRNA海绵而发挥作用。目前共有5项研究^[14, 17-20]^发现circRNA可以对PD-1免疫检查点疗法产生影响，除了circ-UBAP2是通过生信分析发现其可能影响PD-1的表达^[14]^外，其余4个circRNA均通过实验验证了其通过充当miRNA海绵从而发挥作用的具体机制，根据circRNA是否影响PD-1表达可将circRNA分为直接作用（circRNA-002178^[17]^和circ-0001068^[18]^）与间接作用（circ-0020710^[19]^和circFGFR1^[20]^）两类。

直接作用是指circRNA通过一系列复杂机制作用影响到PD-1的表达，以来自肺腺癌细胞的circRNA-002178为例，研究团队证明了癌细胞可以分泌含有circRNA-002178的外泌体，这些外泌体将circRNA-002178转运至CD8^+^ T细胞中并显著富集。通过分析发现circRNA-002178与miR-28-5p之间有两个可以用于结合的结合位点，因此circRNA-002178在T细胞中可以充当miR-28-5p的miRNA海绵从而降低miR-28-5p的水平。既往有研究^[17]^发现miR-28-5p可以通过与T细胞中PD-1的3′UTR相结合从而抑制PD-1的表达，因此降低的miR-28-5p会使PD-1表达升高，故最终的结果表明高表达的circRNA-002178可以促进PD-1的表达。与circRNA-002178类似，来自卵巢癌细胞的circ-0001068也是通过外泌体被转运至T细胞并通过两个结合位点与miR-28-5p结合，降低的miR-28-5p导致与PD-1的结合减少从而促进PD-1的表达^[18]^，进而促进了肿瘤的免疫逃逸。

间接作用是指circRNA并不影响PD-1的表达，而是通过减少免疫细胞浸润等方式形成免疫抑制微环境从而降低PD-1免疫检查点疗法的疗效。有研究^[21]^发现circ-0020710在黑色素瘤中可以通过提供结合位点从而作为miR-370-3p的miRNA海绵与miR-370-3p相结合，通过分析还发现CXC趋化因子配体12[chemokine (C-X-C motif) ligand 12, CXCL12]是miR-370-3p的下游靶标，故通过实验证明了circ-0020710可以通过海绵作用使miR-370-3p表达降低进而导致CXCL12的表达上调。CXCL12又可以招募免疫抑制细胞使细胞毒性T淋巴细胞（cytotoxic lymphocyte, CTL）耗竭，最终导致CTL的浸润减少形成免疫抑制微环境，而包括CTL浸润减少在内的多种免疫抑制机制往往会导致PD-（L）1抑制剂治疗无效。因此作者团队通过实验最终证实了circ-0020710可以通过circ-0020710/miR-370-3p/CXCL12轴形成免疫抑制微环境从而降低PD-1免疫检查点疗法的疗效，同时还通过实验证明了将circ-0020710/CXCL12轴的抑制剂与PD-1抑制剂联用可以提高PD-1治疗的疗效^[19]^。与circ-0020710类似，2019年一项关于非小细胞肺癌（non-small cell lung cancer, NSCLC）的研究^[20]^表明circFGFR1可以通过作为miR-381-3p的miRNA海绵从而上调CXCR4的表达，而既往研究^[22, 23]^发现CXCR4可以阻止CTL的浸润以及导致PD-1抑制剂耐药，故上调的CXCR4可导致免疫抑制微环境最终使circFGFR1发挥免疫抑制作用，此外作者团队还通过回顾性分析证明了高表达circFGFR1的患者对PD-1免疫抑制剂的抗药性更强。

### 1.3 CircRNA影响PD-L1的表达

PD-L1是一种I型跨膜糖蛋白，由1个IgV区、1个IgC区、1个跨膜疏水区和一个细胞质尾部结构域组成^[24]^。PD-L1在不同类型的肿瘤细胞中的表达水平不同，但是在大部分恶性肿瘤中高表达，同时表达水平也与患者的预后紧密联系^[25]^。PD-L1在与免疫细胞的PD-1特异性识别结合后，可以抑制淋巴T细胞的活化，从而帮助肿瘤细胞逃避免疫系统的杀伤^[24]^，通过对PD-1或PD-L1的阻断可以激活抗肿瘤免疫进而杀伤肿瘤细胞^[26]^。

目前共有9项circRNA对PD-L1免疫检查点疗法的影响研究，除了has-circ-0000190是通过统计学方法发现其高血浆水平与高血浆PD-L1水平存在联系（*P*=0.028, 3）^[27]^以及circRNA CDR1-AS可能通过调节CMTM6和CMTM4所需转录因子的功能或表达最终影响PD-L1水平^[8]^外，circRNA-002178（肺腺癌）、circ-CPA4（NSCLC）、circ-0000284（NSCLC）、circCHST15（肺癌）、circKRT1（口腔鳞状细胞癌）、circ-KRT6C（大肠癌）和has-circ-0003288（肝细胞癌）均通过实验证明了circRNA提供结合位点，充当miRNA海绵影响下游靶标表达，最终影响PD-L1表达的机制^[17, 28-33]^。以circ-0000284为例，有研究^[29]^发现并证实了circ-0000284的序列中存在可与miR-377-3p结合的结合位点，其可在癌细胞中充当miRNA海绵从而负性调控下游靶标miR-377-3p，此外在寻找miR-377-3p的下游靶标时发现miR-377-3p可以与PD-L1中的3′UTR相结合进而抑制PD-L1的表达，之后通过使用miR-377-3p抑制剂发现miR-377-3p抑制剂对PD-L1的表达有促进作用，因此证实了PD-L1是miR-377-3p的下游靶标。该研究最终通过实验证明了高表达的circ-0000284在NSCLC中可以通过circ-0000284/miR-377-3p/PD-L1轴促进PD-L1的表达，进而促进肿瘤细胞的免疫逃逸作用。

Eri Tanaka的研究团队针对结肠癌中circRNA CDR1-AS的研究^[8]^发现，circRNA CDR1-AS的高表达可导致CMTM4和CMTM6的表达水平升高，其具体机制不明，但目前已有的结果表明CMTM6和CMTM4的表达上调可能是由circRNA CDR1-AS通过调节CMTM6和CMTM4所需的转录因子间接实现，CMTM4和CMTM6是PD-L1重要的调控分子，它们可以维持细胞表面PD-L1的稳定性^[34, 35]^，因此circRNA CDR1-AS的高表达最终导致结肠癌细胞膜表面的PD-L1水平增加。

## 2 CircRNA与过继性免疫治疗

肿瘤过继性免疫治疗的原理主要是将自体或异体的免疫细胞在体外进行处理，培养出有抗肿瘤活性的效应细胞，并回输至肿瘤患者体内从而达到抗肿瘤的目的。这种疗法包括淋巴因子激活的杀伤细胞（lymphokine-activated killer, LAK）、肿瘤浸润淋巴细胞（tumor infiltrating lymphocyte, TIL）、树突状细胞（dendritic cell, DC）、细胞因子诱导的杀伤细胞（cytokine induced killer, CIK）、自然杀伤细胞（natural killer, NK）、T细胞受体（T cell receptor, TCR-T）、嵌合抗原受体（chimeric antigen receptor, CAR-T）等^[36, 37]^，本综述中涉及到的能够对过继性免疫治疗产生影响的circRNA总结于表 2。

**2:** 环状RNA与过继性免疫治疗 CircRNA and adoptive cellular immunotherapy

CircRNA	Tumor	Type	Effect
CircSnx5	-	DC	A key modulator
CircARSP91	HCC	NK	Enhance tumor immunity
Circ-0000977	PC	NK	Inhibit tumor immunity
CircUHRF1	HCC	NK	Inhibit tumor immunity
Has-circ-0007456	HCC	NK	Enhance tumor immunity
PC: pancreatic cancer; DC: dendritic cell; NK: natural killer.			

目前已经发现的能影响过继性免疫治疗的circRNA研究共5项，分别为circSnx5、circARSP91（肝细胞癌）、circ-0000977（胰腺癌）、has-circ-0007456（肝细胞癌）和circUHRF1（肝细胞癌）^[38-42]^。通过研究^[38]^发现circSnx5可以作为微调DC功能的关键调节剂，这表明其可能作为免疫相关疾病的潜在治疗途径。剩余4个circRNA均与NK细胞有关，其中对circARSP91的研究发现其能够加强NK细胞对癌细胞的杀伤以及癌细胞对NK细胞毒性的敏感性，NK细胞杀伤力的增强与circARSP91促进UL16结合蛋白1（UL16 binding protein 1, ULBP1）的表达并帮助NK细胞识别肿瘤细胞有关，但目前circARSP91调控ULBP1表达机制的解释仍不全面^[39]^；Circ-0000977、has-circ-0007456和circUHRF1则均通过作为miRNA海绵的功能来影响NK细胞介导的肿瘤免疫作用^[40-42]^。

一项关于circ-0000977的研究^[40]^发现缺氧可以诱导胰腺癌细胞中circ-0000977的表达显著增加，circ-0000977可以通过提供结合位点充当miR-153的海绵。MiR-153则可以与下游靶标缺氧诱导因子-1α（hypoxia-inducible factor 1-alpha, HIF1α）和解整合素金属蛋白酶10（a disintegrin and metalloproteinase domain 10, ADAM10）结合从而对HIF1A、ADAM10的表达有抑制作用，当circ-0000977过表达时将解除miR-153对HIF1A和ADAM10表达的抑制作用。ADAM10可以增强膜表面MHC I类相关分子A（membrane MHC class I-related molecule A, mMICA）从癌细胞表面脱落转变为可溶性MICA（soluble MICA, sMICA），sMICA又可以和NK细胞的表面活化性受体D（natural killer group 2 member D, NKG2D）结合，从而导致NK细胞反应低下。因此癌细胞表面mMICA的降低和NK细胞上的NKG2D降低可以促进肿瘤的免疫逃逸^[43]^，过表达的circ-0000977最终使胰腺癌细胞更好地逃避NK细胞的杀伤^[40]^。

与circ-0000977类似，has-circ-0007456与circUHRF1分别通过miR-6852-3p/细胞间黏附因子-1（intercellular adhesion molecule-1, ICAM-1）和miR-449c-5p/T细胞免疫球蛋白黏蛋白-3（T cell immunoglobulin and mucin domain 3, TIM-3）途径调控NK细胞介导的抗肿瘤免疫。Has-circ-000745可使miR-6852-3p的表达下调进而使下游靶标ICAM-1的表达上调，既往已有研究^[41]^发现癌细胞表达的ICAM-1可以与NK细胞上的淋巴细胞功能相关抗原-1（lymphocyte function associated antigen-1, LFA-1）结合从而调节NK细胞与肿瘤细胞之间的黏附，因此过表达的has-circ-0007456将会使NK细胞与肿瘤细胞之间的黏附增加从而增强NK细胞介导的抗肿瘤作用，而circUHRF1则通过充当miR-449c-5p的miRNA海绵使miR-449c-5p的表达下调，则TIM-3作为miR-449c-5p的下游靶标表达将会上调，而TIM-3在既往研究中被发现是NK细胞的主要抑制性受体之一，TIM-3的高表达将会使NK细胞介导的抗肿瘤免疫能力降低，因此高表达的circUHRF1将会降低NK细胞介导的肿瘤免疫能力^[42]^。

## 3 CircRNA与肿瘤疫苗

肿瘤疫苗是通过将含有肿瘤特异性抗原（tumor specific antigen, TSA）或肿瘤相关抗原（tumor-associated antigen, TAA）的肿瘤细胞、外泌体（exosomes, EXO）、多肽及核酸序列等转入患者体内以诱发机体自身免疫应答的方法，从而抑制肿瘤生长甚至清除肿瘤达到治疗肿瘤的目的。肿瘤疫苗分为预防性肿瘤疫苗和治疗性肿瘤疫苗，但是肿瘤疫苗的免疫原性比较低，所以常常需要配合合适的免疫佐剂联合应用，来增加诱导免疫应答的成功率，目前有一项研究^[44]^发现circFOREIGN具有激活先天免疫的潜力，它可以有效刺激免疫基因的表达，因此circFOREIGN可以在体内充当疫苗佐剂诱导抗肿瘤免疫。

## 4 CircRNA与溶瘤病毒

溶瘤病毒是一类可以自我复制的肿瘤杀伤型病毒，其在肿瘤细胞内选择性自我复制并杀灭肿瘤细胞，被感染的肿瘤细胞裂解后除了可以释放子代病毒继续感染周围的肿瘤细胞外，还可以释放肿瘤源性抗原以进一步激活免疫反应，从而达到利用抗肿瘤免疫抑制肿瘤进展的目的^[45]^。有研究^[46]^提示溶瘤痘苗病毒介导的抗肿瘤作用可以通过circRNA-103598/miR-23a-3p/白细胞介素-6（interleukin-6）轴来进行调控，circRNA-103598高表达的甲状腺乳头状癌感染溶瘤痘苗病毒后所表现出的抗肿瘤作用比circRNA-103598低表达的甲状腺乳头状癌更好。

## 5 总结和展望

CircRNA是近些年来研究的热点，其在肿瘤方面的研究方向主要集中在作为肿瘤标志物、影响肿瘤进展转移及预后上，在与肿瘤免疫治疗相关方向上的研究仍比较少。CircRNA具有异常稳定、高度保守且具有组织表达特异性的特性，且circRNA往往在肿瘤等疾病中异常表达，因此其在肿瘤标志物领域中的前景十分广阔，例如has-circ-0000190在一项关于胃癌的研究^[47]^中被认为是比癌胚抗原（carcinoembryonic antigen, CEA）和糖类抗原19-9（carbohydrate antigen 19-9, CA19-9）更加优异的新型胃癌诊断标志物。液体活检是一种全新的、十分具有前景的非侵入型的检测方法，这种方法可以用于分析循环肿瘤DNA（circulating tumor DNA, ctDNA）、循环肿瘤细胞等。CtDNA是一种由于肿瘤细胞的死亡裂解而被释放到血液循环中的DNA小片段，它可以在很多种癌症患者血液中被检测到并且具有一定的特异性与灵敏性，因此可以通过液体活检检测患者血液中的ctDNA以便诊断肿瘤或监测其进展^[48]^。与ctDNA类似，很多肿瘤表达的circRNA可以通过外泌体被分泌至血液中，并且相比较于分泌circRNA的肿瘤细胞，外泌体中的circRNA更加稳定且富集，这使得circRNA可以与ctDNA一样，在血液中可以充当肿瘤标志物被液体活检检测到，从而用于检测肿瘤和预测免疫治疗疗效^[49]^。

除了可以作为肿瘤标志物外，circRNA在肿瘤免疫治疗相关方向中也已经展现出了应用前景。免疫治疗是近些年来具有非常广阔前景的治疗角度和研究方向，虽然目前在免疫检查点抑制剂疗法上已经取得了突破性的进展，但是除此以外的其他免疫疗法仍然处于前期研究状态，暂时无法在临床上大规模应用。单就免疫检查点抑制剂疗法而言，该疗法也仅仅是对一部分患者效果明显，因此还需要进一步的研究以寻找共同特征以用于指导患者的治疗方案选择与用药。在免疫检查点疗法中，circRNA不仅可以作为标志物用于预测使用免疫检查点抑制剂的患者的疗效^[27]^、指导患者用药，还可以通过使用对应的circRNA抑制剂调控其表达从而获得更好的疗效^[19]^。针对除了免疫检查点疗法的其他免疫治疗方法而言，通过调控circRNA有希望提高人体免疫系统对肿瘤的免疫作用。通过基础与临床研究的进一步结合，circRNA有望在以肺癌为首的多种肿瘤的免疫治疗中开辟新的道路，为患者提供新选择，使患者获得更好的生活质量和更长的生存时间；同时也可以推动精准医疗的进展，为免疫治疗提供新的方向。
